# New strategies and therapies for the prevention of heart failure in high‐risk patients

**DOI:** 10.1002/clc.23839

**Published:** 2022-07-05

**Authors:** Michael M. Hammond, Ian K. Everitt, Sadiya S. Khan

**Affiliations:** ^1^ Department of Medicine Northwestern University Feinberg School of Medicine Chicago Illinois USA; ^2^ Department of Preventive Medicine Northwestern University Feinberg School of Medicine Chicago Illinois USA

**Keywords:** heart failure, machine learning, primary prevention, risk prediction, social determinants

## Abstract

Despite declines in total cardiovascular mortality rates in the United States, heart failure (HF) mortality rates as well as hospitalizations and readmissions have increased in the past decade. Increases have been relatively higher among young and middle‐aged adults (<65 years). Therefore, identification of individuals HF at‐risk (Stage A) or with pre‐HF (Stage B) before the onset of overt clinical signs and symptoms (Stage C) is urgently needed. Multivariate risk models (e.g., Pooled Cohort Equations to Prevent Heart Failure [PCP‐HF]) have been externally validated in diverse populations and endorsed by the 2022 HF Guidelines to apply a risk‐based framework for the prevention of HF. However, traditional risk factors included in the PCP‐HF model only account for half of an individual's lifetime risk of HF; novel risk factors (e.g., adverse pregnancy outcomes, impaired lung health, COVID‐19) are emerging as important risk‐enhancing factors that need to be accounted for in personalized approaches to prevention. In addition to determining the role of novel risk‐enhancing factors, integration of social determinants of health (SDoH) in identifying and addressing HF risk is needed to transform the current clinical paradigm for the prevention of HF. Comprehensive strategies to prevent the progression of HF must incorporate pharmacotherapies (e.g., sodium glucose co‐transporter‐2 inhibitors that have also been termed the “statins” of HF prevention), intensive blood pressure lowering, and heart‐healthy behaviors. Future directions include investigation of novel prediction models leveraging machine learning, integration of risk‐enhancing factors and SDoH, and equitable approaches to interventions for risk‐based prevention of HF.

## 1 INTRODUCTION

**Figure 1:**
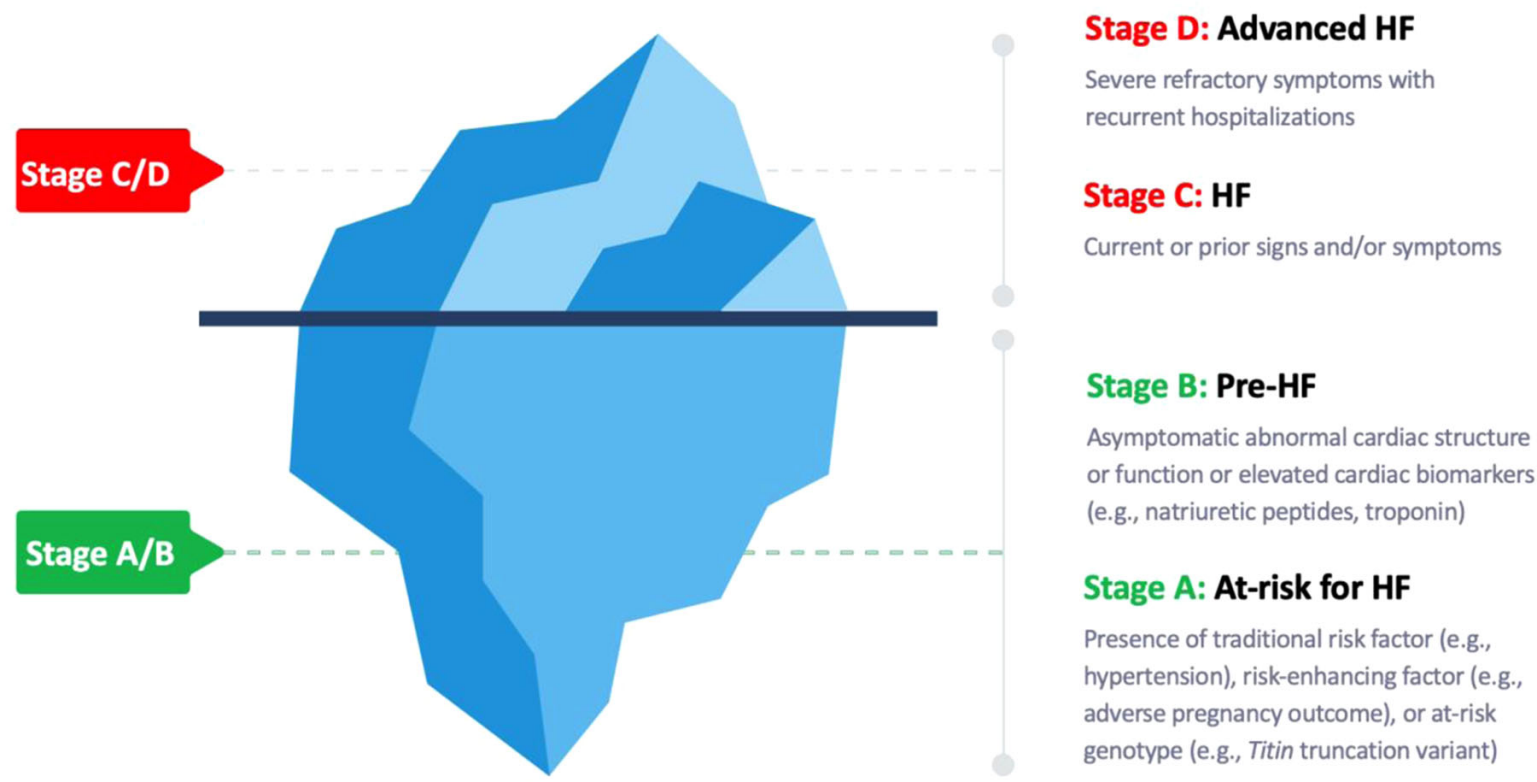
Refocusing on the primary prevention of heart failure. The large proportion of patients with Stage A/B below the surface who are at risk for progression to Stage C/D represents an important target of prevention strategies at the population‐ and individual level. HF, heart failure.

Heart failure (HF) affects about 26 million people worldwide and is an important cause of cardiovascular morbidity and mortality.^1^, ^2^, ^3^ In the United States, about 6.2 million people are affected by HF with a projected prevalence to exceed eight million by 2030.^4^ In addition, healthcare expenditures related to HF are expected to rise to $69.7 billion by 2030.^5^ Due to the increasing prevalence of cardiovascular risk factors (e.g., obesity, hypertension, diabetes),^6^, ^7^ more people are now living at risk of HF. Recent trends show that cardiovascular mortality related to HF has increased in the past decade with the greatest increases among younger adults.^8^ Similarly, increases in HF hospitalizations and readmissions have been observed. Therefore, targeted approaches based on risk assessment are urgently needed to prevent or slow the rise in HF‐related burden. The recent universal classification of HF classifies the stages of HF as stage A: patients at risk of HF but without clinical signs or symptoms of HF; stage B: patients without HF but with abnormal heart structural and function; stage C: patients with signs or symptoms of HF; and stage D: patients with severe advanced signs or symptoms of HF refractory to therapy.^9^ Based on this classification (Figure 1), the population at‐risk (prevalence of stage A or B HF) is much larger than those with clinical signs or symptoms (stage C or D).^10^, ^11^, ^12^ In addition, once a patient progresses from stage A or B to stages C and D, the best outcome is to achieve remission despite optimal guideline‐directed medical treatment.^9^ It is therefore important to focus on primary prevention of HF in patients at stages A and B, where more interventions can prevent or delay progression to later stages.

**Figure 2:**
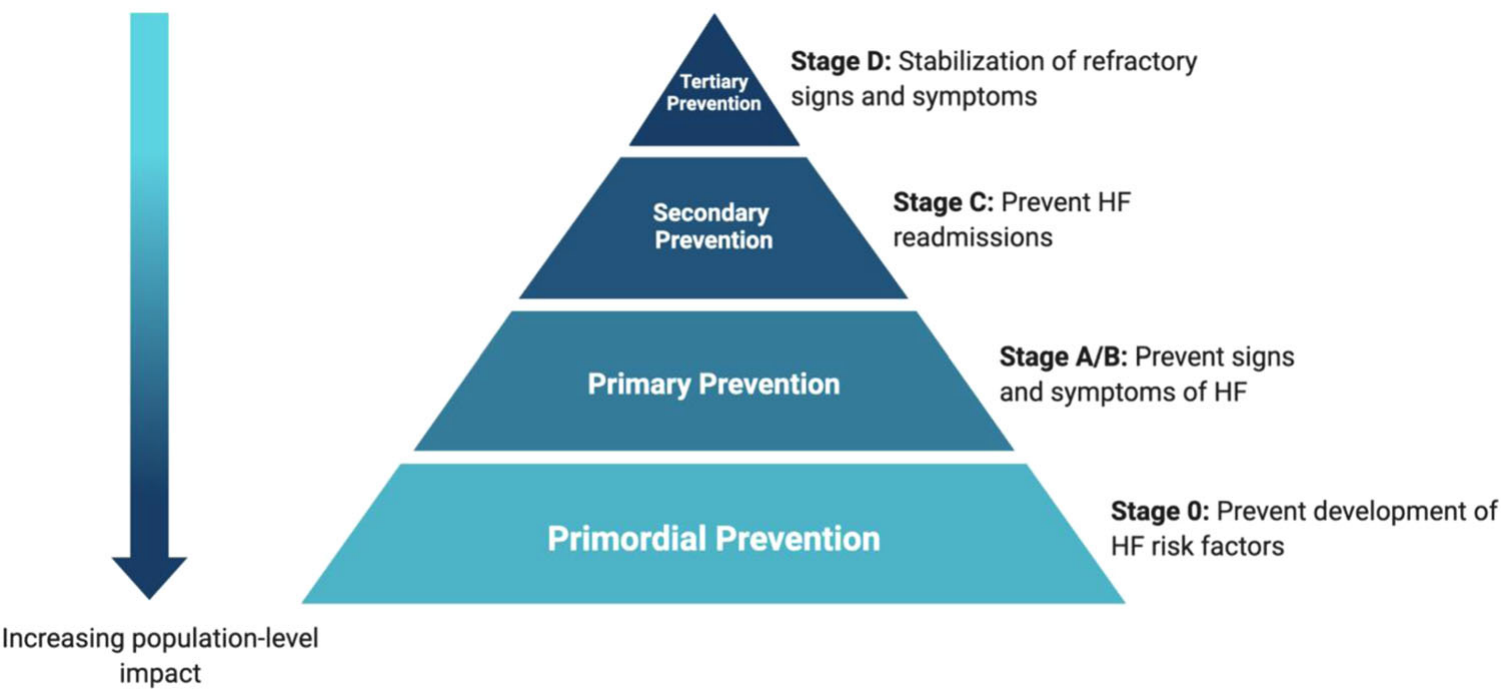
The public health pyramid of stages of prevention applied to heart failure. With each subsequent upstream step in prevention from tertiary to primordial, there is increasing impact at the population level. HF, heart failure.

Strategies to prevent HF are critical to lower the prevalence of HF. From a population‐level perspective, the most effective prevention interventions identify and target high‐risk populations before they develop a disease condition. The four main levels of prevention for public health (Figure 2) include (1) primordial prevention, which focuses on preventing the development of risk factors; (2) primary prevention, which focuses on preventing the onset of disease in people at high risk; (3) secondary prevention, which focuses on preventing recurrence of disease‐related events in people with known disease condition; and (4) tertiary prevention, which focuses on preventing the progression of clinical disease or development of complications in people with a known disease condition.^13^ Contemporary secondary and tertiary HF prevention efforts focus on the reduction of residual risk in patients with stages C and D HF, respectively, who represent a smaller proportion of patients compared to those with stage A and B HF.^14^ Although guideline‐directed medical therapies are increasingly available for HF with reduced ejection fraction (HFrEF), prognosis remains dismal with 50% survival at 5 years.^15^ Further, few effective disease‐modifying therapies currently exist for patients with HF with preserved ejection fraction (HFpEF), which is becoming the most common HF subtype. Therefore, prevention efforts urgently need to also shift upstream to primary and primordial prevention of HF, before irreversible myocardial changes that cause symptomatic HF begin. By focusing on primary and primordial prevention, more people at risk of HF may be prevented from progression to Stages C and D, which may be the “point of no return.” While we often discuss curative therapy in managing malignancy, once signs and symptoms of HF develop (Stage C and D), the target becomes remission and stabilization, and no curative therapies for HF are currently available. As a result, risk prediction to target prevention of HF, particularly for HFpEF, is a critical next step to improve outcomes.

## 2 HF RISK PREDICTION TOOLS

### 2.1 Short‐term multivariable risk prediction models

Although early identification of risk factors is key for primary prevention of HF, patients in Stages A and B are heterogeneous, and therefore identification may be difficult. Despite well‐established data on causal risk factors for HF (e.g., obesity, hypertension, diabetes, tobacco use),^16^, ^17^ models using single (or individual) risk factors may fail to discriminate between many patients who have different combinations of risk factors, and therefore markedly different absolute risk for HF. For example, not all patients with diabetes have a similarly high risk of HF. To address this heterogeneity in risk, several risk prediction tools have been developed that combine risk factor levels to facilitate preventive interventions based on absolute risk levels. Whereas risk‐based prevention (matching the intensity of prevention with the absolute risk of the individual) is widely accepted in the primary prevention of atherosclerotic cardiovascular disease, no such prevention paradigm currently exists for HF in clinical practice. Earlier risk prediction models developed in single cohorts, such as the Framingham Heart Study, Atherosclerosis Risk in Communities Study, the Health ABC, and the Atherosclerosis Risk in Communities Study included demographic and clinical risk factors for HF.^18^ However, each of these models had variable performance in external validation studies.^6^, ^19^

More recently, the Pooled Cohort Equations to Prevent HF (PCP‐HF) tool was developed using routinely collected risk factor data (age, body mass index, systolic blood pressure, treatment for hypertension, fasting glucose, treatment for diabetes, total cholesterol, high‐density lipoprotein cholesterol, smoking status, and QRS duration on electrocardiography [ECG]) from 23 541 participants enrolled in five cohort‐based studies with at least 12 years follow‐up.^20^ The model derivation sample included adults aged 30–79 years and performed well in internal and external validation in identifying and discriminating risk of HF.^20^ In external validation, the model had good discrimination and calibration in diverse samples.^20^ Furthermore, the PCP‐HF model has also been validated in real‐world data from electronic health records (with and without ECG QRS as a predictor to enhance generalizability),^21^ and has been extended to large, nationally representative samples in the United States, Europe, and in Israel.^22^ ^,^ ^23^

Based on the available data, the 2022 American College of Cardiology/American Heart Association/Heart Failure Society of America (ACC/AHA/HFSA) Guideline for the Management of HF recommended, for the first time, consideration of validated multivariable scores (e.g., PCP‐HF) to estimate the risk of incident HF in the general population with a class of recommendation 2a and level of evidence B‐NR.^24^ This advances the paradigm of prevention of HF moving beyond focus on individual risk factor control and towards absolute risk.^25^, ^26^, ^27^

Patients in Stage A may have a lower risk of HF compared to those at Stage B. However, the association between HF risk factors and HF is variable (i.e., different patients at Stage A may have different risks of HF because of the different risk factors that they have). Risk prediction models have also been developed to calculate risk in specific high‐risk populations that are known to have significantly increased risk of HF.^26^ Risk prediction models have been developed for the elderly,^6^, ^28^ patients with hypertension,^29^ patients with diabetes,^30^, ^31^ and patients with chronic kidney disease.^32^ For example, Sahle et al.^29^ developed a tool for 10‐year prediction of incident HF in elderly people with hypertension using routinely collected demographic and clinical data. However, the model was not externally validated.^29^ Additionally, risk prediction models have been developed to discriminate between risk of HFpEF and HFrEF.^33^

Traditional HF risk prediction models^6^, ^18^, ^19^, ^20^, ^28^ have largely been based on traditional statistical modeling with Cox proportional hazard regression, which faces limitations, including the inability to utilize nonlinear outcomes. Advances in machine learning allows for the inclusion of large amounts of multidimensional data, are well equipped to handle missing data on covariates, and can adjust for nonlinear interactions between covariates. Machine learning models also offer the ability to continuously update models as new covariates and data become available in additional cohorts. Machine learning‐derived models have shown the ability to predict adverse events in population‐based studies^34^, ^35^; one such machine learning‐derived model developed by Segar et al.^36^ using clinical, laboratory, and biomarker data demonstrated superior performance to multiple traditional HF risk prediction models. A key future step for machine learning‐derived risk assessment of groups at high risk of HF will be the integration of machine learning‐based models into the electronic health records to allow for better data capture and, ideally, to enable models to provide real‐time estimates of a patient's HF risk. As a result, these models could enable prospective identification of individuals at increased risk for HF (and HF subtypes) for targeted screening (e.g., biomarkers, echocardiography), prevention strategies (e.g., sodium glucose co‐transporter 2 inhibitors [SGLT2i]), and recruitment of high‐risk phenotypes for randomized clinical trials focused on HF prevention.

### 2.2 Long‐term HF risk prediction

The majority of risk prediction tools have focused only on short‐term (5–10 year) risk of HF.^20^, ^21^, ^22^, ^29^ However, because risk of HF across the lifetime is substantial (ranging between 20% and 46% at age 45 years), increases with age, and varies based on the prevalence of cardiovascular risk factors, long‐term risk prediction tools are needed. Importantly, an individual who may be at low short‐term risk but high long‐term risk for HF may not be identified with short‐term risk prediction alone. Therefore, focusing only on short‐term risk represents a key opportunity for prevention to intervene on long‐term risk. Recently, our group developed the first long‐term risk prediction model to estimate 30‐year risk of HF.^37^ The models had strong predictive ability in women and men with all *C*‐statistics greater than 0.80.^37^

### 2.3 Laboratory and imaging biomarkers for risk prediction

Contemporary HF risk prediction tools primarily rely on well‐studied traditional risk factors for the development of incident HF, including the presence or absence of hypertension, coronary artery disease, and diabetes; body mass index; and tobacco use. However, there has been growing interest in incorporating blood‐, electrocardiographic (ECG)‐, and echocardiographic (TTE)‐based biomarkers that may enhance risk prediction, particularly among individuals without established cardiovascular disease.^38^, ^39^ Elevated levels of routinely collected biomarkers of neurohormonal stress, myocardial injury, systemic inflammation, as well as the presence of left ventricular hypertrophy indicate the presence of systemic derangement early in the course of HF disease progression, and have been associated with a higher risk of incident HF among healthy community‐dwelling adults.^38^, ^40^, ^41^, ^42^

Cardiac troponin (cTn) is a marker of myocardial injury and N‐terminal pro‐B‐type natriuretic peptide (NT‐proBNP) and B‐type natriuretic peptide (BNP) are biomarkers of myocardial neurohormonal stress that are detectable in the general population and associated with risk of adverse cardiovascular events.^41^, ^43^ These biomarkers have demonstrated some ability to predict incident HF, and currently comprise the most commonly utilized biomarkers in risk models.^19^, ^28^, ^31^, ^44^, ^45^ However, routine use of these biomarkers is not currently widespread in clinical practice to identify HF risk despite guideline recommendations for BNP‐based prevention. Additional biomarkers that have shown predictive value for the development of incident HF include high‐sensitivity C‐reactive protein (hs‐CRP),^31^, ^40^, ^44^ a nonspecific marker of systemic inflammation; plasminogen activator inhibitor (PAI)‐1, d‐dimer, and fibrinogen^44^ which represent thrombotic and fibrinolytic pathways; galectin‐3 and soluble interleukin‐1 receptor‐like 1 (sST2)^44^, ^46^ which indicate tissue fibrosis; and cystatin‐C^44^ which reflects renal dysfunction. Commonly measured biomarkers, including cTn, BNP, and hs‐CRP appear to be more strongly correlated with the development of HFrEF than HFpEF.^47^ A substantial evidence base exists supporting the role of BNP/NT‐proBNP and cTn as diagnostic and prognostic markers of acute and chronic HF;^48^, ^49^, ^50^, ^51^, ^52^, ^53^ however, the recent emergence of sST2 and galectin‐3 as prognostic markers led to their inclusion in the 2017 HF guidelines for additive risk stratification.^25^ Routine use of blood‐based biomarkers to assess risk of incident HF is complicated by changes in circulating levels of cTn and BNP/NT‐proBNP across the lifespan—these markers are influenced by age,^54^ renal function,^55^ body composition,^56^, ^57^ menopause,^58^ and pregnancy.^59^, ^60^ Further research is needed to elucidate specific patient populations for whom routine use of blood‐based biomarkers in the use of HF risk prediction may be of most benefit that account for cost and scalability.

Several ECG findings have been associated with incident HF in adults without existing CAD, including QRS prolongation >120 ms^61^ and left ventricular hypertrophy^62^, ^63^ (defined using a variety of criteria). These ECG findings represent underlying changes to myocardial structure, which may reflect left ventricular systolic dysfunction, and suggest patients who have transitioned from Stage A to Stage B. A prolonged QRS, in particular, has also been associated with increased mortality in Stage C HF with improved outcomes with cardiac resynchronization therapy in this subset of symptomatic patients.^25^, ^64^ As a result, QRS prolongation and ECG criteria for left ventricular hypertrophy have been integrated into several HF predictive risk scores.^19^, ^20^ However, routine ECG is not recommended in all adults by the United States Preventive Services Task Force (USPSTF), which leads to barriers in implementation of such risk scores.

Similarly, TTE provides noninvasive measures of subclinical systolic and diastolic dysfunction that may allow early detection of patients transitioning from Stage A to Stage B. Left ventricular hypertrophy, particularly when accompanied by systolic dysfunction or diastolic dysfunction, has been associated with increased risk of incident HF.^65^, ^66^ Global longitudinal strain can detect subtle systolic dysfunction before a reduction in ejection fraction occurs, and has been used to predict progression of HF in Stage A/B individuals across the lifespan.^67^, ^68^, ^69^ Future outcome studies are needed to better define the role of TTE and advanced cardiac imaging in the prevention of progression of HF in high‐risk individuals, both as a screening mechanism and as tools to assess the impact of risk factor modification and medical therapy on incident HF, particularly with cost‐effectiveness in mind.

## 3 RISK‐ENHANCING FACTORS ASSOCIATED WITH HF

**Figure 3:**
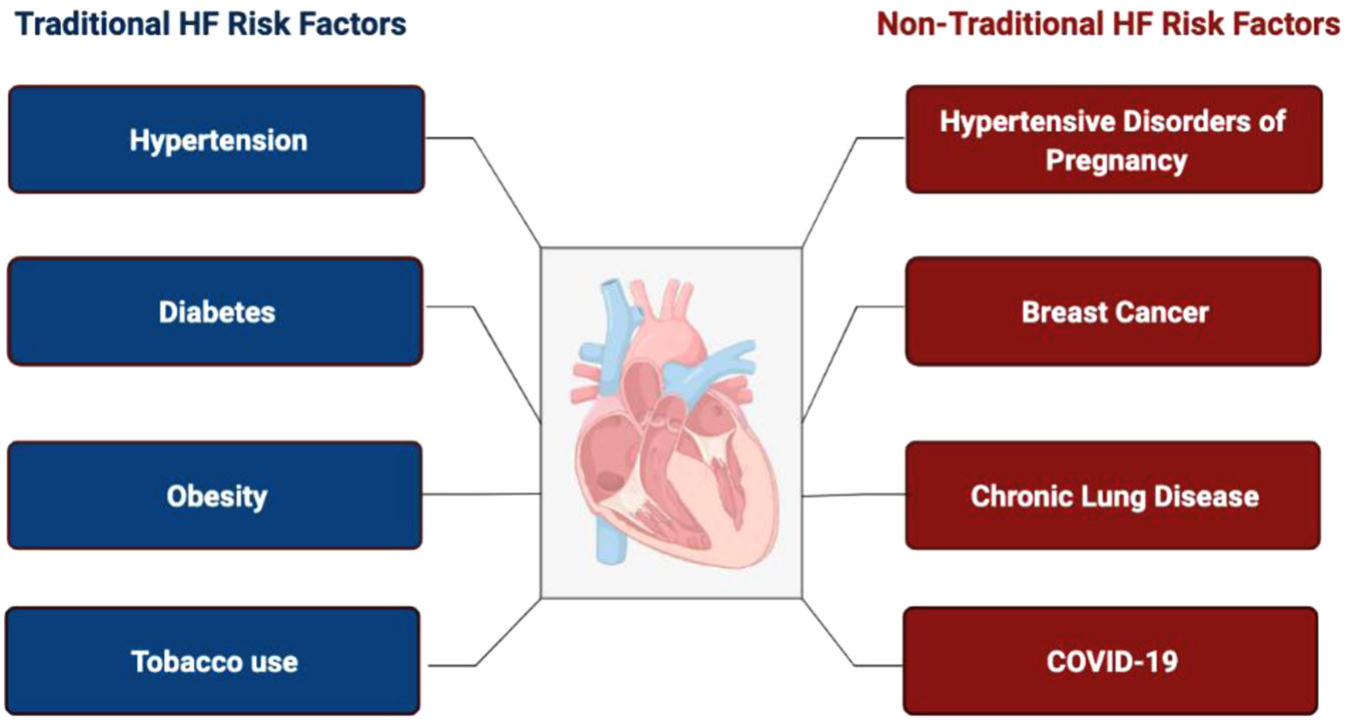
Traditional and nontraditional risk factors for heart failure. Only half of lifetime risk of heart failure is captured by traditional risk factors. Emerging risk‐enhancing factors should be incorporated into personalized strategies for prevention of heart failure and include (but are not limited to) hypertensive disorders of pregnancy, breast cancer, chronic lung disease, and Covid‐19. HF, heart failure.

Although multivariable HF risk prediction models discriminate HF risk and stratify individuals based on estimates of risk, they may underestimate risk in people without traditional HF risk factors. The use of risk‐enhancing factors to personalize atherosclerotic cardiovascular disease risk may similarly be applied to HF risk prediction to personalize HF risk. About 50% of population attributable fraction of HF is associated with the presence of traditional risk factors, specifically obesity, hypertension, diabetes, hyperlipidemia, and smoking.^70^ Risk‐enhancing factors are nontraditional risk factors that have not classically been included in HF prediction tools, but may provide an opportunity to personalize the individual‐level approach to HF prevention (Figure 3). Some representative examples are discussed here, which include hypertensive disorders of pregnancy, breast cancer, chronic lung disease, and Covid‐19.

### 3.1 Hypertensive disorders of pregnancy

Pre‐eclampsia, which affects about 2%–8% of all pregnancies, is associated with a fourfold higher maternal risk of future HF.^71^, ^72^ Women who develop pre‐eclampsia during pregnancy are at higher risk of future hypertension and diabetes,^73^, ^74^ which are known risk factors of HF. Although the mechanism by which pre‐eclampsia increases the risk of HF is not well‐established, the factors that predispose women to pre‐eclampsia are similar to risk factors for HF,^16^, ^75^, ^76^ and therefore may be targeted for pre‐eclampsia prevention, and subsequent HF prevention (e.g., optimizing dietary quality, increasing physical activity, maintaining a healthy body mass index).

### 3.2 Breast cancer

Breast cancer is the most common malignancy among women in the United States,^77^ and associated with an increased risk for HF.^78^, ^79^ Additionally, several chemotherapy agents for breast cancer such as doxorubicin, cyclophosphamide, anastrozole, and trastuzumab increase risk for HF.^80^, ^81^ The 5‐year survival after initial breast cancer diagnosis is about 90%, with about three million breast cancer survivors in the United States who are at increased risk of HF.^82^ Measurement of left ventricular ejection fraction before, during, or after chemotherapy for breast cancer may allow for early detection of cardiovascular toxicity related to treatment for early intervention.^81^ Since risk factors for breast cancer and HF are similar (e.g., smoking, obesity, sedentary lifestyle) and shared mechanisms may increase risk for both cancer recurrence and HF (e.g., inflammation, angiogenesis, clonal hematopoiesis of indeterminate potential),^82^ incorporation of breast cancer and cardiotoxic chemotherapy regimens as risk‐enhancing factors in HF risk prediction may help individualize and optimize prevention strategies for both HF and cancer.

### 3.3 Chronic lung diseases

In 2010, about 384 million people were affected by chronic obstructive pulmonary disease (COPD),^83^ and by 2030, COPD is projected to be the fourth leading cause of death worldwide.^84^ COPD and HF commonly coexist and are associated with significantly higher morbidity and mortality, particularly among older adults.^85^, ^86^, ^87^ Beyond the concurrence of COPD and HF that is attributable to shared upstream risk factors (e.g., smoking, aging), COPD is independently associated with an increased risk of HF that may be, in part, mediated by inflammation.^88^, ^89^ Even in the absence of symptomatic lung disease, subclinical impairments in lung health are associated with adverse cardiac remodeling. Therefore, it may be important to include chronic lung disease as a risk‐enhancing factor to improve personalization of HF risk prediction. However, data are needed on the timing and associated mechanisms of the transition from heart and lung health to disease across the life course.

### 3.4 Covid‐19 infection

In the United States, about 63 million cases of Covid‐19 and about 840 000 deaths related to Covid‐19 were recorded as of January 2022.^90^ Although Covid‐19 was initially thought to predominantly affect the respiratory system, it rapidly became evident that acute and postacute infection with Covid‐19 affects multiple organs, including the heart with evidence of biomarker elevation in many hospitalized patients.^91^ Among 243 hospitalized patients with acute respiratory distress syndrome (ARDS) due to Covid‐19 who required intubation and mechanical ventilation from a single academic center, 51% had troponin levels above the upper limit of normal.^92^ When compared with patients with ARDS without Covid‐19, rates of biomarker elevation were lower after adjustment for age and comorbidities suggesting observed myocardial injury reflects critical illness rather than direct viral injury. In patients with pre‐existing HF (either with reduced or preserved ejection fraction), Covid‐19 is associated with an increased risk for HF hospitalization^93^ and in‐hospital death.^94^ Emerging data also suggest increased short‐ and long‐term risk of incident HF after Covid‐19 infection. One study from Germany of 100 individuals who were at least 2 weeks post‐Covid‐19 diagnosis (median [interquartile range] 71 [64–92] days) demonstrated evidence of myocardial inflammation on cardiac magnetic resonance imaging in 60% of people.^95^ Furthermore, recent evidence suggests that Covid‐19 increases the risk of long‐term cardiovascular outcomes, including HF (even among patients who were not hospitalized during the acute phase of Covid‐19 infection). In a nationwide cohort study from the Department of Veteran Affairs, 153 760 individuals with Covid‐19 had a higher risk of HF at 12 months (HR: 1.72 [1.65, 1.80]) compared with 5 637 647 controls.^96^ More studies are needed to improve understanding of both short‐ and long‐term complications of Covid‐19 on heart health., However, due to the high prevalence of Covid‐19 and higher risk of severe Covid‐19 infection among those with HF risk factors (e.g., obesity, hypertension),^90^ incorporation of history or current Covid‐19 infection as a risk enhancing factor in HF risk prediction models may help to better stratify risk in HF prevention and inform patient management after surviving Covid‐19.

## 4 SOCIAL DETERMINANTS OF RISK FOR HF

In recent years a growing body of research has examined the association between a multitude of social, demographic, economic, and environmental domains, including race, ethnicity, access to health care and health insurance, public health infrastructure, neighborhood environment, air pollution, economic stability, and education and risk of incident HF. Racial disparities in both the prevalence and incidence of HF are well‐established; the prevalence of HF is greater among Black individuals than among non‐Hispanic White individuals,^97^ and Black individuals develop HF at younger ages than White individuals.^98^ However, additional social determinants have an established and consistent association with incident HF, including living in an area with few healthcare services, lack of health insurance, low educational attainment, increased exposure to particulate air matter, and low annual income.^17^, ^99^ Meta‐analysis of the independent contribution of socioeconomic status to risk of incident HF found a 62% increase in HF risk associated with socioeconomic deprivation by any measure of SES, including income, education, occupation, or area‐level measures of neighborhood deprivation.^100^ Furthermore, there is also evidence to suggest that multiple socially determined vulnerabilities have a cumulative effect on HF risk within the same individual, particularly in individuals under 65 years of age independent of traditional risk factors.^101^ A sum of socially determined vulnerabilities may substitute as an additional risk enhancer in patients who are at increased risk of incident HF, especially among younger individuals. Importantly, social and political systematic changes implemented with the aim of reducing social, environmental, and economic disparities provide a significant opportunity to address upstream risk factors before patients become adversely affected by downstream effects, including poverty, poor education, inadequate housing, decreased access to nutritious food, and inadequate access to healthcare resources.^99^ Efforts to address social determinants of risk must simultaneously address structural and systemic barriers (e.g., racism) that contribute to HF risk and may provide the highest‐impact opportunities to conduct both primordial and primary HF prevention across the spectrum of risk.

## 5 GENETIC RISK MARKERS FOR HF: OVERLAP WITH CARDIOMYOPATHY

HF is a heterogeneous syndrome that represents the terminal clinical pathway of most cardiovascular diseases and manifests as an array of phenotypes. There is also significant diversity mirrored in the genetic architecture of HF, which can range from multiple genetic or epigenetic low‐penetrance loci that are modified by environmental factors to monogenic HF syndromes resulting from a single rare disease‐causing or pathogenic variant (e.g., *Titin‐*truncating variants).^102^ Genetic cardiomyopathies comprise a small proportion of overall incident HF, although this varies by age and population. Among pediatric patients, a familial monogenic origin has been identified in 26%–40% of patients, while among adults with idiopathic dilated cardiomyopathy the proportion of familial disease is approximately 30%.^103^, ^104^, ^105^ Susceptibility to HF is also heritable as a complex trait, with heritability estimated to be ~18%.^106^ Cardiovascular research has made rapid advancements in elucidating the cardiac epigenome, and in the last several years epigenome‐wide associations have linked epigenetic loci with clinical HF in living patients utilizing a multiomics approach, in which genomic, epigenomic, transcriptomic, proteomic, and microbiome data are combined in analysis.^107^, ^108^, ^109^ These methods offer significant potential for the development of precision medicine approaches to identification of risk and risk‐based prevention of HF.^110^ Using an integrative approach, it may eventually be possible to combine an individual's multi‐OMIC data into an individual risk profile, which, when combined with phenotypic clinical and sociodemographic data incorporated from the electronic health record, may enhance prediction of incident HF as well as HF outcomes utilizing machine learning techniques and artificial intelligence.

## 6 PREVENTION STRATEGIES FOR HIGH‐RISK INDIVIDUALS

### 6.1 Heart‐healthy lifestyle modifications

Lifestyle factors are associated with the risk of HF.^111^, ^112^ Based on evidence from multiple clinical trials and observational studies, the AHA recommends seven health factors to define cardiovascular health: Life's Simple 7.^113^ These factors include: (1) not smoking, (2) optimal levels of physical activity, (3) healthy diet quality, (4) normal body mass index, (5) optimize cholesterol, (6) control fasting blood glucose, and (7) optimize blood pressure.^113^ Smoking increases risk for HF.^114^ Moreover, people who have never smoked have a lower risk of HF compared with former smokers.^115^ Engaging in >150 min/week of moderate‐intensity physical activity or >75 min/week of high‐intensity activity is associated with a lower risk of HF.^116^, ^117^ Similarly, Mediterranean and Dietary Approaches to Stop Hypertension (DASH) diets are associated with lower cardiovascular risk and better cardiovascular health.^118^, ^119^, ^120^ Age‐specific interventions that target frailty in older adults may also have benefit in prevention of HF.

### 6.2 Intensive blood pressure lowering

Patients with hypertension have a higher risk for HF compared with normotensive patients.^121^ In both patients with and without known cardiovascular disease, blood pressure lowering is associated with lower risk of adverse outcomes.^122^ Based on evidence of greater benefits of lower blood pressure, ACC/AHA guidelines recommend blood pressure lower than 130/80 mmHg for optimal cardiovascular risk, but treatment is initiated when blood pressure >140/90 mmHg.^123^ The systolic blood pressure intervention trial (SPRINT) trial documented that intensive blood pressure lowering to a systolic pressure <120 mmHg decreased risk for future HF compared with <140 mmHg,^124^, ^125^ While there is growing evidence for the effectiveness of intensive blood pressure lowering in the prevention of HF, intensive blood pressure lowering can also lead to an increase in serious adverse events. Therefore, maximizing the benefit and minimizing adverse effects by estimating HF risk may guide selection for intensive BP lowering. A recent post hoc analysis of the Systolic Blood Pressure Intervention Trial (SPRINT) demonstrated greater risk reduction in those with the highest baseline risk (low risk: HR: 0.86 [95% CI: 0.29, 2.56]; intermediate risk: 0.54 [0.23, 1.30]; high risk: 0.45 [0.23, 0.86]).^126^ Despite the benefits of intensive blood pressure lowering in HF prevention, there remains ongoing debate regarding the comparative effectiveness of various antihypertensive therapies. Emerging therapies for HF treatment, such as sacubitril‐valsartan, have not been studied in the prevention of HF but may offer benefits related to both BP lowering as well as reverse cardiac remodeling. A study by Sciarretta et al.^127^ showed that diuretics, angiotensin‐converting enzyme inhibitors, and angiotensin receptor blockers (ARBs) were more effective than calcium channel blockers for HF prevention.^127^

### 6.3 Biomarker‐based prevention

Two landmark trials that focused on biomarker‐based prevention of HF: the STOP‐HF (St. Vincent Screening to Prevent Heart Failure)^128^ and PONTIAC (NT‐proBNP selected prevention of cardiac events in a population of diabetic patients without a history of cardiac disease)^129^ led to a guideline‐based recommendation for natriuretic peptide‐based prevention. Both of these trials focused on screening with either BNP or NT‐proBNP to identify patients at risk for HF. Participants randomized to the intervention were then referred for intensive preventive therapies, such as up‐titration of renin–angiotensin–aldosterone system antagonists and beta‐blockers, which led to lower risk of incident subclinical or clinical HF (systolic or diastolic). However, widespread implementation of BNP‐based screening continues to be limited given the cost and lack of clarity in which patients may benefit from biomarker testing. Alternative strategies include sequential biomarker testing with initial assessment of risk using multivariable risk prediction models that only require readily available clinical factors, such as the PCP‐HF. Among those at intermediate risk, biomarker‐based testing may improve reclassification. Future studies investigating the utility of combining risk factor‐based models with biomarker‐based models are needed.

### 6.4 Mineralocorticoid receptor antagonists (MRA)

Despite the beneficial effects of aldosterone in normal physiologic regulation of body sodium, potassium, and water regulation, aldosterone can also negatively affect the heart, including inducing inflammation, stiffening of vessels, and stimulation of fibrosis in myocardium.^130^, ^131^, ^132^ Aldosterone may therefore play an important role in the pathogenesis of HF. As such, MRAs are associated with improved outcomes in patients with HFrEF.^133^, ^134^, ^135^ In the Treatment of Preserved Cardiac Function Heart Failure With an Aldosterone Antagonist (TOPCAT) trial, although spironolactone did not effectively reduce risk of the composite outcome of adverse cardiovascular events (death from cardiovascular causes, aborted cardiac arrest, or hospitalization for the management of HF), it reduced the risk of HF hospitalization.^136^ Despite established benefits of MRAs in HF, there have been no large‐scale randomized control trials to study the effects of MRAs in primary prevention of HF.

### 6.5 Sodium‐glucose co‐Transporter‐2 inhibitors (SGLT2i)

SGLT2i are novel drugs that lower blood glucose by decreasing the rate of glucose reabsorption and increasing glucose excretion.^137^ SGLT2i reduces risk of adverse cardiovascular events in people with diabetes.^138^, ^139^, ^140^ SGLT2i also reduced the risk of cardiovascular death or HF hospitalizations in patients with and without diabetes with chronic kidney disease, HFrEF, and HFpEF.^141^, ^142^, ^143^, ^144^ The benefits of SGLT2i have also been observed in patients who are at high risk of HF but do not have a known history of HF and suggest a role for SGLT2i in the primary prevention of HF. As a result, SGLT2i's have been proposed for use in a risk‐based algorithm for the prevention of HF,^145^ akin to the role of statins in atherosclerotic cardiovascular disease risk prevention.

### 6.6 Telemedicine in HF prevention

Significant rural–urban disparities exist in cardiovascular health and HF mortality. One potential approach to focus on equitable prevention strategies is telemedicine, which includes home telemonitoring and telephone‐supported monitoring. Telemedicine has been demonstrated to be effective in reducing mortality and hospital admission rates among people with HF.^146^, ^147^ The prevalence of telemedicine interventions has grown rapidly as a result of the Covid‐19 pandemic, when medical systems rapidly transitioned to noncontact care delivery methods for ambulatory care as social lockdowns, quarantine, and perceived risk of infection resulted in decreased contact between patients with HF and medical contacts.^148^, ^149^ Future studies are needed to evaluate the effect of telemedicine in HF prevention.

## 7 FUTURE DIRECTIONS AND CONCLUSIONS

Given the growing burden of HF hospitalization and mortality, strategies are urgently needed to focus on the primary prevention of HF before the onset of clinical signs and symptoms. The goal will be to reduce the number of people who develop Stage C or Stage D HF who have a dismal prognosis. Intervening earlier during the risk stage with preventive strategies may offer the greatest yield before irreversible damage has occurred. This will require an understanding of which populations are at highest risk of developing HF to match the intensity of the preventive intervention with the absolute risk of the individual. Several risk prediction tools have been developed, including the PCP‐HF model that is well‐validated in multiple international populations and endorsed by the 2022 multisociety HF Guidelines in the United States.^24^ There is a wide array of genetic, biologic, clinical, and socioeconomic markers of enhanced risk, which may enhance personalized risk prediction when combined with traditional risk factor levels. Furthermore, machine learning and multi‐OMIC approaches offer further opportunities to identify novel mechanisms and refine risk estimates. Once individuals at risk are identified, implementation gaps that persist in achieving optimal risk factor levels need to be addressed. Lastly, future research is necessary to determine whether specific high‐risk individuals may benefit from medical therapy, including MRA or SGLT2i, for the primary prevention of incident HF.

## CONFLICT OF INTEREST

The authors declare no conflicts of interest.

## Data Availability

Data sharing is not applicable to this article as no new data were created or analyzed in this study.
